# Efficient Inactivation of Symbiotic Nitrogen Fixation Related Genes in *Lotus japonicus* Using CRISPR-Cas9

**DOI:** 10.3389/fpls.2016.01333

**Published:** 2016-08-31

**Authors:** Longxiang Wang, Longlong Wang, Qian Tan, Qiuling Fan, Hui Zhu, Zonglie Hong, Zhongming Zhang, Deqiang Duanmu

**Affiliations:** ^1^State Key Laboratory of Agricultural Microbiology, Huazhong Agricultural UniversityWuhan, China; ^2^College of Life Science and Technology, Huazhong Agricultural UniversityWuhan, China; ^3^Department of Plant, Soil, and Entomological Sciences and Program of Microbiology, Molecular Biology, and Biochemistry, University of IdahoMoscow, ID, USA

**Keywords:** *Lotus japonicus*, CRISPR/Cas9, symbiosis, genome editing, leghemoglobin, nitrogen fixation

## Abstract

The targeted genome editing technique, CRISPR/Cas9 system, has been widely used to modify genes of interest in a predictable and precise manner. In this study, we describe the CRISPR/Cas9-mediated efficient editing of representative SNF (symbiotic nitrogen fixation) related genes in the model legume *Lotus japonicus* via *Agrobacterium*-mediated stable or hairy root transformation. We first predicted nine endogenous *U6* genes in Lotus and then demonstrated the efficacy of the *LjU6-1* gene promoter in driving expression of single guide RNAs (sgRNAs) by using a split yellow fluorescence protein (YFP) reporter system to restore the fluorescence in Arabidopsis protoplasts. Next, we chose a customized sgRNA targeting *SYMRK* (symbiosis receptor-like kinase) loci and achieved ~35% mutagenic efficiency in 20 T0 transgenic plants, two of them containing biallelic homozygous mutations with a 2-bp deletion near the PAM region. We further designed two sgRNAs targeting three homologous leghemoglobin loci (*LjLb1, LjLb2, LjLb3*) for testing the possibility of generating multi-gene knockouts. 20 out of 70 hairy root transgenic plants exhibited white nodules, with at least two *LjLbs* disrupted in each plant. Compared with the constitutively active CaMV 35S promoter, the nodule-specific *LjLb2* promoter was also effective in gene editing in nodules by hairy root transformation. Triple mutant knockout of *LjLbs* was also obtained by stable transformation using two sgRNAs. Collectively, these studies demonstrate that the CRISPR/Cas9 system should greatly facilitate functional analyses of SNF related genes in *Lotus japonicus*.

## Introduction

The bioavailability of soil organic nitrogen is one of the most important limiting factors to plant growth and crop yield. In modern agricultural practice, farmers heavily rely on the use of nitrogen and phosphate fertilizers to improve the crop yield (Rogers and Oldroyd, 2014). However, excessive use of chemical fertilizers will inevitably lead to depletion of natural resources and cause significant pollution of soils, groundwater and lake systems (Galloway et al., 2008). Nitrogen fertilizer, i.e., ammonia, is manufactured by the Haber Process that combines atmospheric nitrogen with hydrogen derived mainly from natural gas under high pressure, high temperature and in the presence of catalysts. Because of the economic and environmental costs of ammonia produced in this fashion, finding alternatives to this chemical fertilizer is critical for sustained growth of the agricultural industry (Charpentier and Oldroyd, 2010). Some bacteria and Archaea species can directly convert atmospheric nitrogen into ammonium through the prokaryote-exclusive enzyme nitrogenase, which is composed of two component metalloproteins, the reductase component (Component II) and the catalytic component (Component I; Seefeldt et al., 2009). A small percentage of these nitrogen-fixing microorganisms, i.e., rhizobia, have evolved the ability to establish symbiotic associations with host plants (Masson-Boivin et al., 2009). Within the nitrogen-fixing nodules of legumes, bacteroids utilize carbohydrates derived from plant photosynthate and, in return, provide fixed nitrogen compounds to help host plants grow when the environmental nitrogen supply is limited (Oldroyd et al., 2011). Detailed investigation of the molecular mechanisms of rhizobia recognition and nodule organogenesis in legumes may provide promising targets for engineering cereal crops with the capability to accommodate nitrogen-fixing bacteria intracellularly to fix their own nitrogen and thus reduce the use of N fertilizer in the future (Charpentier and Oldroyd, 2010; Geurts et al., 2016).

*Lotus japonicus* is a commonly used model legume species in the SNF (symbiotic nitrogen fixation) research community, together with *Medicago truncatula* and *Glycine max*. A few mutant libraries of Medicago and soybean have been constructed by EMS chemical induction (Perry et al., 2009), *Tnt1*-insertion (Tadege et al., 2008; Cui et al., 2013) and *LORE1*-insertional mutagenesis (Fukai et al., 2012; Urbanski et al., 2012). Several SNF-related genes have been identified in Lotus, including the two nodulation factor receptors (*NFR1* and *NFR5*) (Radutoiu et al., 2003), the nuclear cation channels (*POLLUX, CASTOR*) (Imaizumi-Anraku et al., 2005), the calcium-dependent and calmodulin-dependent protein kinase (*CCaMK*) (Tirichine et al., 2006), the Lotus histidine kinase1 (*LHK1*) (Murray et al., 2007; Tirichine et al., 2007) and the nodule inception transcription factor (*NIN*) (Schauser et al., 1999).

The symbiosis receptor-like kinase *SYMRK*, with an extracellular leucine-rich-repeats, a transmembrane domain and an intracellular kinase domain, is essential for symbioses of legumes with both rhizobia and arbuscular mycorrhizal fungi by participating in the symbiotic signal transduction of fungal or bacterial perception to rapid symbiosis related gene expression (Stracke et al., 2002). Ectopic expression of *SYMRK* or its dominant active allele could initiate nodule formation in the absence of rhizobia, while loss-of-function *symrk* mutants were unable to form root nodules and arbuscular mycorrhiza in Lotus (Stracke et al., 2002; Ried et al., 2014). Lotus also encode three leghemoglobin genes (*LjLb1, LjLb2, LjLb3*). RNAi knock-down of the *LjLbs* genes revealed their essential functions in establishing low free-oxygen concentration but high energy status (ATP/ADP) within nodules for effective SNF (Ott et al., 2005, 2009). To gain deeper insights into the biological functions and genetic relationships of these genes, yeast two hybrid and other biochemical approaches have been employed to identify the interacting partners of these key proteins (Chen et al., 2012). However, the lack of corresponding mutants in many legumes and the lengthy procedure needed for obtaining homozygous mutants have significantly hampered progress in understanding the molecular mechanisms of nodule development and SNF in legumes.

With the goal of knocking-out specific target genes in various model organisms, a series of precise gene editing techniques have been developed, such as zinc finger nucleases (ZFNs) (Lloyd et al., 2005), transcription activator-like effector nucleases (TALENs) (Li et al., 2012) and clustered regularly-interspaced short palindromic repeats (CRISPR) (Mali et al., 2013). Compared with the more complex ZFN and TALEN systems, the CRISPR/Cas9 system is comprised of only two simple parts, a CRISPR-associated protein 9 nuclease (Cas9) and an engineered single guide RNA (sgRNA) that specifies the target site in the genome. The synthetic sgRNA can form a complex with Cas9 protein and accurately guide the complex to a specific 20 bp DNA sequences where the HNH nuclease domain and the RuvC-like domain of Cas9 protein cut two opposite strands of the target DNA 3 base-pairs upstream of a 3-nucleotide PAM motif (i.e., NGG). This process induces double strand breaks (DSBs) of genomic DNA, which are then repaired through homologous recombination (HR) or non-homologous end joining (NHEJ) (Doudna and Charpentier, 2014).

The simplicity and wide applicability of various optimized CRISPR/Cas9 systems (Cong et al., 2013; Mali et al., 2013) has made it a rapidly adopted tool for gene editing in a variety of plants, including Arabidopsis (Feng et al., 2014; Zhang et al., 2015), rice (Shan et al., 2013), tobacco (Li et al., 2013), soybean (Li et al., 2015; Sun et al., 2015), sorghum (Jiang et al., 2013), tomato (Brooks et al., 2014), and potato (Wang et al., 2015). Besides allowing rapid creation of mutant libraries for investigating the functions of specific genes in model organisms, CRISPR/Cas9 system can also accelerate generation of multiplexed genome modifications of homologous genes or gene families in a much shorter time than conventional breeding techniques (Ding et al., 2016).

In this study, we demonstrated the applicability of the CRISPR/Cas9 system to efficiently target single and multiple SNF genes in stable transgenic Lotus plants or by hairy root transformation. As an effective supplement to current EMS, *Tnt1* and *LORE1* retrotransposon mutant libraries, this technique has the potential to make genome editing a routine practice in Lotus and should significantly shorten the time needed to acquire mutant plants containing multiple combinations of disrupted genes.

## Materials and methods

### Vector construction

The 102 nt ncRNA sequence of *Arabidopsis thaliana* U6-26 snRNA gene (NCBI accession: X52528.1) was used as a query to BLAST the *Lotus japonicus* genome database (http://www.kazusa.or.jp/lotus/). Nine putative U6-snRNA genes were identified and we carried out multiple sequence alignment among these genes (Figure S1). The *LjU6-1* promoter was PCR-amplified (SgRNA-Kpn1-Spe1-F and U6-sgRNA-fusion-R) from wild type *Lotus japonicus* MG20 genomic DNA and the sgRNA region was PCR-amplified (U6-sgRNA-fusion-F and SgRNA-Xba1-Sal1-R) from the pBlueScript SK+-AtU6-26 sgRNA vector. The fusion fragment was then subcloned into pBlueScript SK+-AtU6-26 sgRNA vector (Feng et al., 2013) by replacing the AtU6-26 promoter element. The *Bbs*I site in the *LjU6* promoter region was mutated (primers Bbs1-mut-F and Bbs1-mut-R) before plasmid construction to facilitate downstream cloning. We used the web tool CRISPR-P (Lei et al., 2014) (http://cbi.hzau.edu.cn/crispr/) to select specific single-guide RNA sequences. The *LjU6*-sgRNA fragments between *Kpn*I and *Sal*I sites, together with the *Kpn*I and *Eco*RI double-digested 2 × 35S-Cas9, were cloned into the pCAMBIA1300 vector. To construct the tissue-specific expression vector, the 2 × 35S constitutive promoter (SK+-35S-Cas9 vector) was replaced with *LjLb2* promoter (1363 bp) using the *Xho*I and *Kpn*I enzyme sites and the hygromycin-resistance gene (pCAMBIA1300 vector) was replaced with sGFP gene (XhoI-sGFP-Fand XhoI-sGFP-R) using the *Xho*I enzyme site. Primers and vectors used in this study are listed in supplemental Tables S1, S2, respectively.

### Arabidopsis protoplast preparation and YF-FP homologous recombination reporter assay

The transient YF-FP-HR reporter assay was performed according to the method described by Feng et al. (2013). The Arabidopsis mesophyll protoplasts isolation and reporter plasmids transfection were conducted by a standard procedure (Yoo et al., 2007). 12~16 h after DNA transfection, fluorescence signals were analyzed by the “Microscopic Analysis” described below.

### Microscopic analysis

Microscopic analysis was performed using the Nikon SMZ18 and the Olympus FV1000 confocal laser-scanning fluorescence microscope. Imaging services were provided by the microscopic analysis facility of the State Key Laboratory of Agricultural Microbiology at Huazhong Agricultural University.

### Transient gene expression analysis in *Nicotiana benthamiana*

All the vectors were electroporated into *Agrobacterium tumefaciens* strain EHA105 and were used for transient expression in *N benthamiana* according to the protocol described by Sparkes et al. (2006). After 36 to 48 h post-infiltration, leaf tissues were harvested, immediately frozen in liquid nitrogen and stored at −80°C until use. Total RNA was isolated from tobacco leaves using TRIzol Reagent (Invitrogen) following the recommended protocol. One microgram total RNA was reverse transcribed using EasyScript one-step gDNA Removal and cDNA Synthesis Super Mix kit (TransGen Biotech, China). PCR amplification was performed using gene specific forward primers (YF-FP-sgRNA-F, SYMRK-sgRNA-F, Lb-sgRNA1-F, Lb-sgRNA2-F) and RT-sgRNA-R primer (sequences of these primers can be found in Table S1) under the following conditions: 95°C for 5 min; 31 cycles (94°C for 30 s, 58°C for 30 s, 72°C for 15 s); and 72°C for 5 min.

The Cas9 nuclease expression assay was performed according to the following procedure. About 100 mg of tobacco leaves were finely pulverized in liquid nitrogen with a cold mortar and pestle. The fine powder was transferred into 1.5 mL eppendorf tubes using a cold stainless-steel lab spoon and incubated with 400 μL protein extraction buffer containing 50 mM Tris-HCl (pH 7.4), 150 mM NaCl, 1% Triton X-100, 1% NP-40, 0.1% SDS, 1 × protease inhibitor cocktail (Roche) and 2 mM PMSF (phenylmethylsulfonyl fluoride, Sigma). The tubes were then shaken vigorously for 30~60 s until the powder was fully dissolved, incubated at 4°C for 30 min and then centrifuged at 13,000 *g* at 4°C for 15 min. About 300 μL supernatant was transferred into a new 1.5 mL tube and the protein concentration was quantified with the Pierce™ BCA Protein Assay Kit (Thermo Fisher). Equal amount of total proteins (~30 μg) were mixed with 4 × SDS loading buffer (40% Glycerol, 240 mM Tris/HCL pH 6.8, 8% SDS, 0.01% bromophenol blue, 10% β-mercaptoethanol), boiled for 10 min and were then separated on a 10% SDS polyacrylamide gel and transferred to PVDF membrane. Membranes were blocked with 5% non-fat dry milk in Tris-buffered saline (TBS, pH 7.2~7.4) for 2~4 h at room temperature. Blocked membranes were washed with TBST (TBS containing 0.1% Tween-20) for 5 min and incubated with anti-FLAG antibody (Sigma, 1:2000 dilution) in TBST for 2 h at room temperature. Membranes were then washed four times with TBST. HRP-conjugated goat anti-mouse secondary antibody (1 mg/ml, Proteintech Group Inc., China) was diluted 1:5000 in TBST and incubated with the membranes for 1 h at room temperature. Membranes were washed as above and chemiluminescence signals were detected with ChemiScope 6300 imaging system in accordance with the recommended instructions (Shanghai Clinx Science Instruments Co., Ltd, China).

### Plant transformation

*A. tumefaciens*-mediated stable transformation of *Lotus japonicus* MG20 was conducted by a standard procedure (Tirichine et al., 2005) with minor modifications. In brief, seeds were sterilized and germinated on plates with MS (Murashige & Skoog) medium (4.33 g/L Basal Salt Mixture (Sigma), 0.103 g/L vitamin mixtures (Catalog number M7150, Sigma), 30 g/L Sucrose (Sigma), 8 g/L Agar (Sigma), pH 6.0) for 4 d in the dark at 22°C, followed by 3 d under 16 h light/8 h darkness photocycle in a growth cabinet. The explants of the split hypocotyl were infected with *A.tumefaciens* EHA105 containing the appropriate plasmids for ~30 min. The co-cultivation plates (10 pieces of sterile filter paper soaked with 1/10 × Gamborg's B-5 liquid medium, Sigma) was incubated for 7 d at 21°C in the dark. After that, the explants were transferred to regeneration medium (Gamborg's B-5 medium, 3.1 g/L Basal Salt Mixture (Sigma), 1 × Vitamin solution (Catalog number G1019, Sigma), 20 g/L Sucrose (Sigma), 4 g/L Phytagel (Sigma), pH 5.5) containing 0.4 μg/mL 6-BA (Solarbio, China), 0.04 μg/mL NAA (Solarbio, China), 15 μg/mL hygromycin (Roche) and 300 μg/mL timentin (Solarbio, China) for callus formation. The transgenic calli were transferred to fresh regeneration medium every week for 1~2 months. After that, the green calli were constantly transferred to shoot induction medium (Gamborg's B-5 medium) containing 5 mM (NH_4_)_2_SO_4_, 0.4 μg/mL 6-BA (Solarbio, China), 15 μg/mL hygromycin (Roche) and 300 μg/mL timentin (Solarbio, China) until shoots were 2~4 cm long. Individual shoots were transferred to root induction medium (Gamborg's B-5 medium) containing 0.1 μg/mL NAA, 15 μg/mL hygromycin and 300 μg/mL timentin and propagated until roots appeared.

Hairy root transformation of *Lotus japonicus* MG20 using the *A. rhizogenes* strain LBA1334 was described previously (Díaz et al., 2005). LBA1334 carrying the binary plasmids of interest was grown on Luria-Bertani (LB) plate supplemented with 50 μg/mL kanamycin (Teknova, USA) and 25 μg/mL rifampicin (Biosharp, China) for 2~3 days. To synchronize the emergence of hairy roots, 7-day-old Lotus MG20 seedlings were cut at the base of hypocotyls and moved to a conical flask containing resuspended Agrobacterium (final OD_600_~ 0.6). After being soaked for half an hour in the flask, the hypocotyls were transferred onto MS plates (Sigma) and placed in the growth chamber for 5 days. The plants were transferred onto HRE (Hairy Root Emergence, *Lotus japonicus* Handbook, http://link.springer.com/book/10.1007/1-4020-3735-X) medium plates containing 300 μg/mL timentin and grown for 10~15 days at 22°C with 16 h light/8 h darkness and under white light intensity of 60~100 μmol photons m^−2^ s^−1^. Plants were then transferred to nutrition pots filled with vermiculite and perlite (2:1) and grown in a green house at 23°C with 16 h light/8 h darkness and under white light intensity of 60~100 μmol photons m^−2^ s^−1^. When the first true leaf appeared, plants were inoculated with *Mesorhizobium loti* strain MAFF303099 to assay nodulation phenotypes.

### DNA extraction, PCR/RE assay, and sequencing

Genomic DNA was extracted using a method described by Li et al. (2010). PCR amplification was performed using gene specific primers under the conditions mentioned above. PCR products were digested with appropriate restriction enzymes and separated on a 1% agarose gel. Appropriate DNA bands were extracted from the gel, purified and cloned into the pMD19T-simple vector (Takara). Five individual clones were sequenced by Sanger sequencing using gene specific primers. For gene loci with no appropriate restriction enzymes, PCR products were directly sequenced by Sanger sequencing. If mutations were identified, each PCR product was cloned into the pMD19T-simple vector, individual *E. coli* colony was selected and then sequenced to confirm the mutation.

### RT-PCR and qRT-PCR

Five days post inoculation with *M. loti*, total RNA was isolated using Trizol Plants Total RNA Isolation Kit (TransGen Biotech, China) from at least 100 mg fresh root materials (~3 plants). Primescript RT Reagent kit (Takara) was used to synthesize first strand cDNAs. Reverse transcription-PCR (RT-PCR) amplification was performed using gene specific primers (SYMRK-RT-F and SYMRK-RT-R) under the following conditions: 95°C for 5 min; 31 cycles (94°C for 30 s, 58°C for 30 s, 72°C for 1 min 15 s); and 72°C for 5 min.

Real-time quantitative reverse transcription (qRT)-PCR was performed using the SYBR Select Master Mix reagent (Applied Biosystems) under the following conditions: Hold Stage, 50°C for 2 min, 95°C for 10 min; PCR Stage, 40 cycles (95°C for 15 s, 60°C for 1 min); and Melt curve Stage, 60°C to 95°C at the speed of 0.05°C/s. qRT-PCR reactions were performed using an ABI ViiA™ 7 Real-Time PCR System. The expression levels of *LjSYMRK* (with SYMRK-qRT-F and SYMRK-qRT-R primers) were calculated by the 2^−ΔΔCt^ method and normalized against the polyubiquitin gene (*LjUBI*, GenBank accession no. AW720576). For each genotype, at least three biological replicates and three technical replicates were analyzed.

### Leghemoglobin immunoblot analysis

Detailed procedures for protein immunoblot assay have been described in “Transient gene expression analysis in *Nicotiana benthamiana*.” For *Lotus japonicus* leghemoglobin western analysis, in brief, protein samples were harvested 1 month post inoculation. About 100~150 mg fresh nodules were finely pulverized in liquid nitrogen with a cold mortar and pestle. Protein samples were separated on a 12–15% SDS-PAGE gel. Western-blot analysis was performed using primary antibody against soybean leghemoglobin GmLba (a generous gift from Professor Yangrong Cao of Huazhong Agricultural University, China) and HRP-conjugated rabbit anti-goat secondary antibody. As a positive control, Glutathione *S*-transferase (GST) tagged *LjLb3* was expressed in *E. coli* BL21. Cells were disrupted by high pressure homogenization (D-3L; PhD Technology International, MN, USA) and protein was purified by GST column following recommended procedures (Sangon Biotech, China).

## Results

### Engineered CRISPR/Cas9 system restored YFP fluorescence in arabidopsis protoplasts

**Figure 1:**
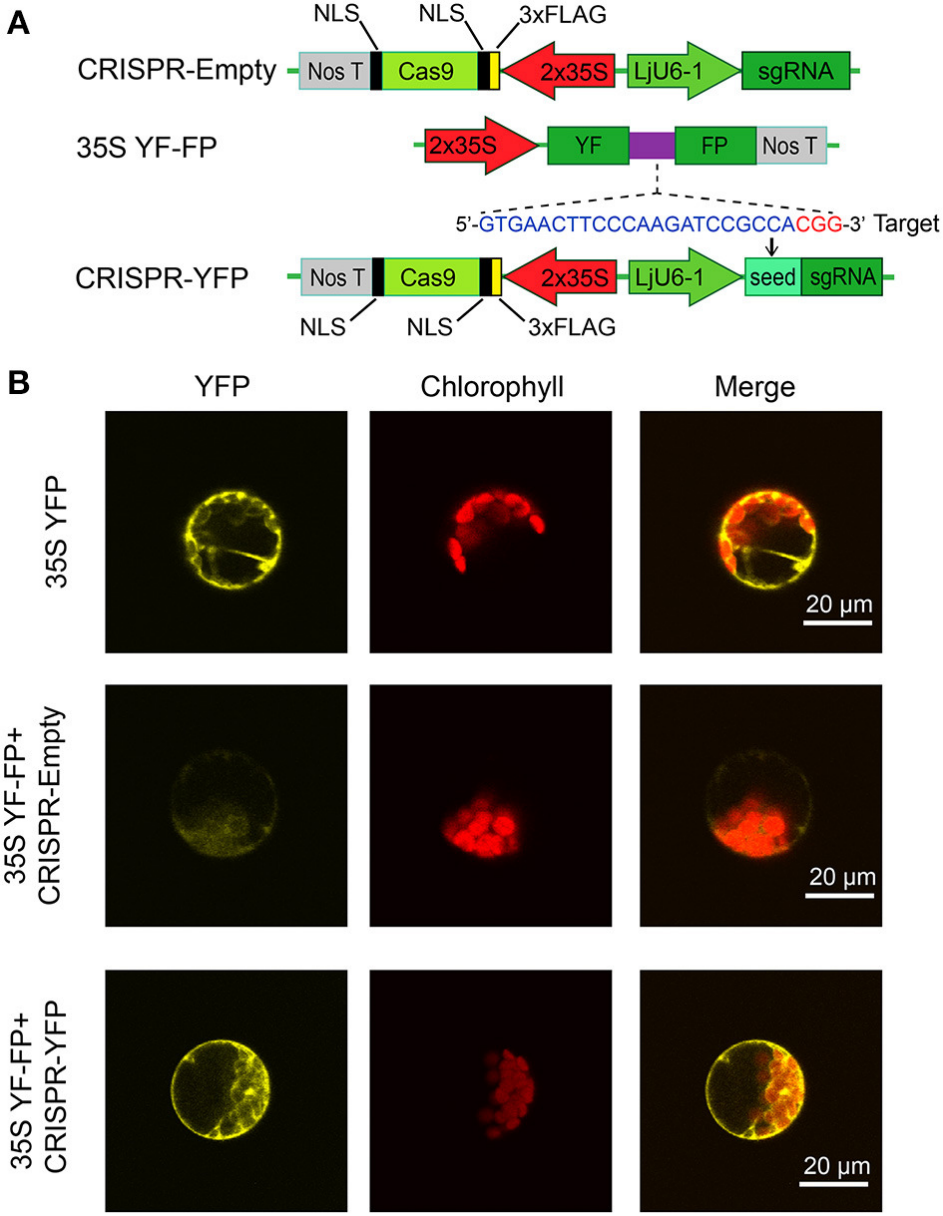
**Validation of the CRISPR/Cas9 system for YFP fluorescence restoration in Arabidopsis protoplast. (A)** Schematic depiction of the CRISPR/Cas9 binary vectors and YF-FP reporter system. The Cas9 cassette was driven by the 2 × 35S promoter, while sgRNAs are controlled by the *LjU6-1* promoter. The 35S YF-FP reporter construct contains two separate fragments of YFP (YF and FP, respectively) with partial overlapping region that can recombine by homologous recombination (HR) when the DNA target sequence is successfully cleaved by the Cas9/sgRNA complex and, thereby, generate a functional YFP gene. The PAM sequence of the sgRNA target is highlighted in red and the sgRNA target is in blue. **(B)** Functionality of the CRISPR/Cas9 system was tested in Arabidopsis protoplasts. The YFP fluorescence (left) and the chlorophyll auto-fluorescence (center) were observed under confocal microscopy and merged (right). The 35S YFP (top panel) was chosen as positive control, while the 35S YF-FP and CRISPR-Empty (middle panel) were co-transformed as a negative control. In the presence of sgRNA targeting YF-FP overlapping region, the HR-based YFP fluorescence restoration was observed (bottom panel).

To achieve efficient targeted gene editing, a single guide RNA (sgRNA) needs to be expressed to direct the Cas9 endonuclease to cleave corresponding DNA targets. Bortesi and Fischer (2015) compared various promoters that have been used to generate sgRNAs in different plants. *U6* or *U3* small nuclear RNA (snRNA) promoters (recognized by RNA polymerase III) were used most frequently. Based on the previous study in Arabidopsis by Feng et al. (2013), we chose the *Lotus japonicus* endogenous *U6* promoter to drive the sgRNA expression. Nine putative *U6*-snRNA genes (Figure S1A) were identified in the Lotus genome based on the Arabidopsis *U6-26* snRNA sequence. Both USE (upstream sequence element) and TATA-like box elements are conserved in the promoter region (Waibel and Filipowicz, 1990), which are indispensable for the transcription of snRNAs. Subsequently, we cloned an ~800 bp promoter region of *LjU6-1* from the genomic DNA of Lotus ecotype MG20 (Miyakojima) (Figure S1B). A *Bbs*I recognition site in the *LjU6-1* gene promoter was mutated (G → C) so that the guide RNA can be inserted between two *Bbs*I sites with annealed oligonucleotides. The CAMV 35S promoter (2 × 35S) was used to express the codon-optimized hSpCas9 (Feng et al., 2013). To test the efficacy of the engineered CRISPR/Cas9 system, we took advantage of a split yellow fluorescent protein (YFP) reporter system, YF-FP, in Arabidopsis protoplasts based on homologous recombination (HR) restoration of the fluorescence (Figure 1A). The 35S YFP and 35S YF-FP + CRISPR-Empty were used as positive and negative controls, respectively. After transient coexpression of the 35S YF-FP and CRISPR-YFP constructs, the YFP signal was readily detected using a confocal laser-scanning fluorescence microscope (Figure 1B). These results demonstrated that *LjU6-1* promoter is functional in driving expression of the sgRNA gene and that the reconstructed CRISPR/Cas9 system is competent in generating precise DNA modification in plant cells.

### CRISPR/Cas9 induced indels of the *LjSYMRK* gene in stable transgenic lotus plants

**Figure 2:**
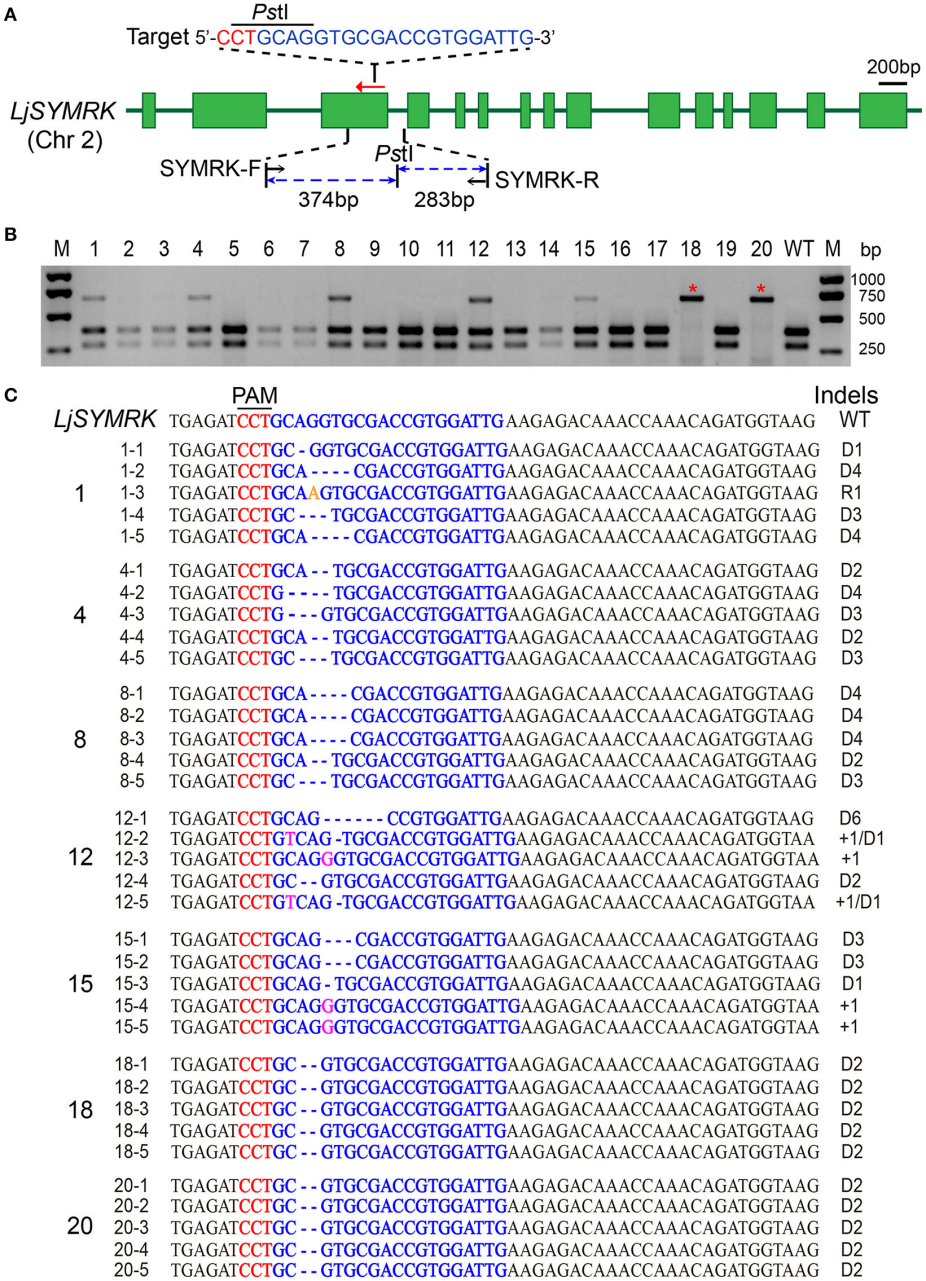
**CRISPR/Cas9 modification of *LjSYMRK* in stable transgenic Lotus plants. (A)** Schematic diagram illustrating the genome structure of the *LjSYMRK* gene. The PAM sequence of the *LjSYMRK* sgRNA target is colored in red and the sgRNA target is in blue. Two primers (SYMRK-F/SYMRK-R) were used to amplify the target region for sequencing confirmation. A *PstI* recognition site (CTGCAG) overlaps the Cas9/sgRNA cleavage site 3 bp downstream of the NGG/CCT PAM sequence. **(B)** Genotyping of 20 T0 transgenic lines by PCR/RE (Polymerase Chain Reaction/Restriction Enzyme) assay. The PCR products were digested with *Pst*I enzyme and produced ~374 and ~283 bp fragments with DNA from WT plants, but an intact ~657 bp PCR product if the *PstI* restriction site was destroyed by Cas9 cleavage and by subsequent erroneous DNA repair. M, DNA Marker. **(C)** Representative indel mutations in the vicinity of the PAM site (colored in red) of the *LjSYMRK* gene. The sgRNA target is highlighted in blue. WT, wide-type control. D1/2/3/4/6, 1 bp/2 bp/3 bp/4 bp/6 bp DNA deletion; +1, 1 bp insertion; R1, 1 bp replacement.

We selected four *Lotus japonicus* symbiosis related genes as target loci, including the symbiosis receptor kinase *LjSYMRK*, and the symbiotic leghemoglobin genes *LjLb1, LjLb2, LjLb3* (GenBank accession numbers: AF492655, AB042716, AB042717, and AB008224, respectively). Gene-specific guide sequences were designed by the web tool CRISPR-P, which allowed us to screen for highly specific editing sites within target DNA sequences and provided off-target prediction simultaneously (Lei et al., 2014). One guide sequence (SYMRK-sgRNA) was designed to target the third exon of *LjSYMRK*, while two guide RNAs were chosen to target the three *LjLb* genes simultaneously (Lb-sgRNA1 and Lb-sgRNA2) (Figures 2A, 4A, respectively). Successful expression of sgRNA and FLAG-tagged Cas9 was confirmed by reverse transcription PCR (RT-PCR) (Figure S2A) and Western blotting (Figure S2B) in the infiltrated tobacco leaves.

The transformed *A.tumefaciens* EHA105 harboring the SYMRK-sgRNA was used for stable transformation of *Lotus japonicus*. Twenty T0 transgenic plants were collected for PCR/RE (restriction enzyme) assay to detect mutations around the target site (Figure 2B). The enzyme-digestion pattern of 13 samples (65%) were the same as wide-type control, possibly suggesting that no mutations occurred in the target site. The other seven samples (35%) showed a mixture of *PstI* digested PCR fragements as well as intact, undigested PCR fragments (lines #1, #4, #8, #12, #15, #18, #20; Figure 2B), with the latter indicating the occurrence of Cas9/sgRNA-dependent mutations in the target site of some *LjSYMRK* alleles. The mixture of both digested and undigested bands suggested heterozygous alleles in the target gene. Interestingly, two of them (lines #18 and #20) were completely resistant to enzyme digestion. Sequencing analysis indicates these two transgenic lines contain homozygous mutations of the two *SYMRK* alleles in the genome, with identical 2 bp deletions at the site of Cas9 DNA cleavage. The 2 bp deletion near the PAM sequence was retained in the transcript and there was no evidence of alternative splicing in the knockout lines (Figure S3A). Quantitative measurement of *LjSYMRK* gene expression by qRT-PCR indicated no significant changes in line #18 and line #20, compared with WT and EMS61 mutant (Figure S3B). Thus, line #18 and #20 are most likely complete knockout in *LjSYMRK* gene because of the 2 bp deletion in exon 3.

PCR products of other 5 putative mutants were also cloned and individually sequenced (Figure 2C). We found that all these 5 plants contained various mutant alleles including diverse deletion, insertion and substitution mutations. Overall, our results demonstrated that the mutation frequency of the Lotus *LjSYMRK* gene using just one sgRNA was notably high (35%, 7 out of 20 T0 transgenic lines).

**Figure 3:**
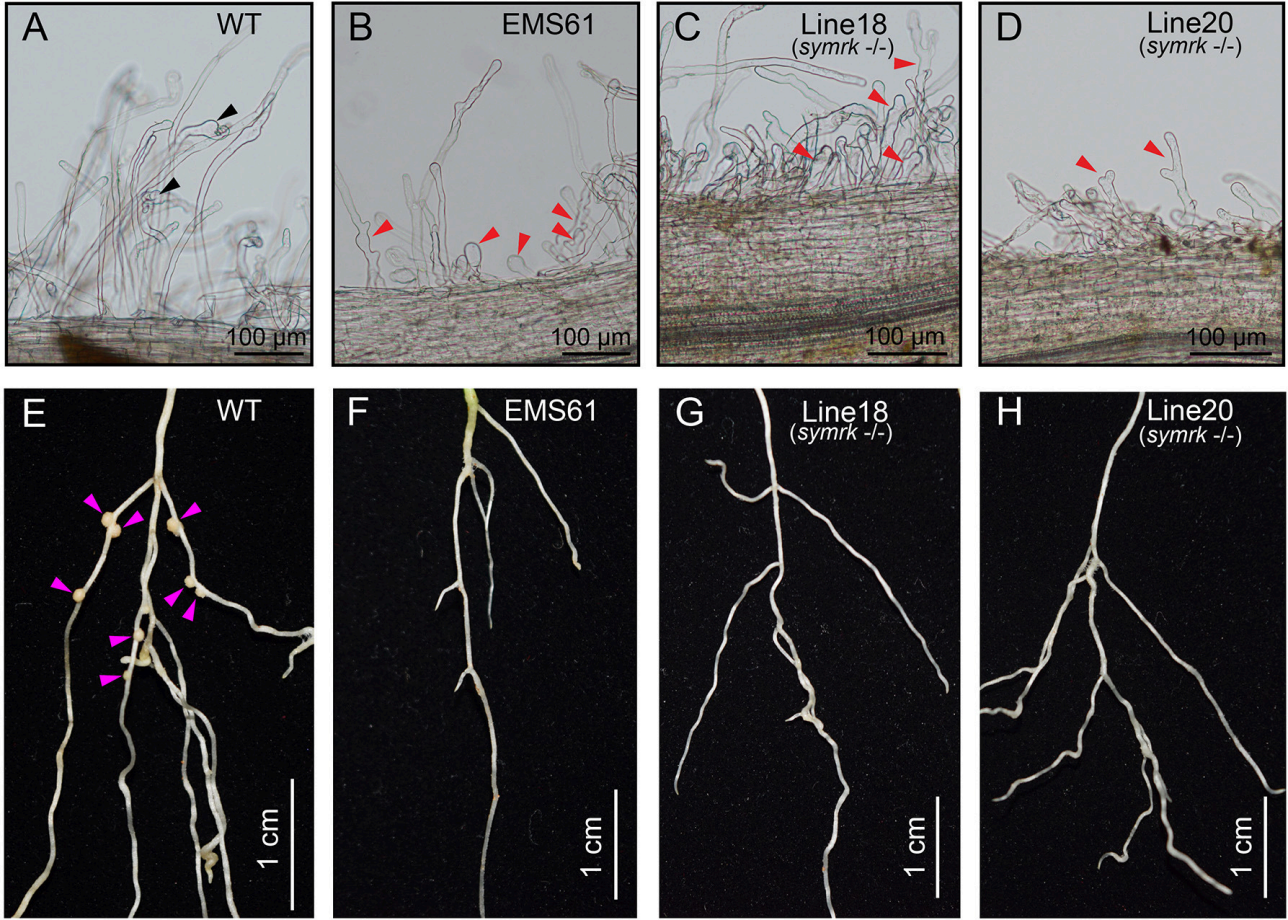
**Symbiotic phenotypes of *symrk* mutants. (A–D)** Root hair responses of wild type MG20 plants **(A)**, the *symrk* loss of function mutant EMS61 **(B)**, and the T1 progeny of stable transgenic lines #18 and #20 **(C,D)** after 3~6 days post inoculation with *Mesorhizobium loti* strain MAFF303099. The EMS61 and *symrk* mutants formed inflated and biforked root hairs (red arrowheads in **B–D**), whereas WT root hairs exhibited tip swelling and entrapped rhizobia that led to the formation of infection chamber (**A**, black arrowheads). **(E–H)** Nodulation phenotypes of corresponding plants after 12 days post inoculation. None of the mutant lines could form nodules **(F–H)**, whereas WT plants formed typical nodule primordia and nodules (**E**, purple arrowheads). 10~15 plants were analyzed for each genotype.

We next compared the symbiotic phenotype of the T1 progeny from line #18 and line #20, together with the *LjSYMRK* mutant EMS61 (Stracke et al., 2002) and MG20 WT plants. The mutant EMS61 was generated by EMS mutagenesis and contained a nonsense mutation in the SYMRK kinase domain. Seedlings were grown in a nutrient-poor soil medium containing vermiculite and perlite (volume ratio 2:1) supplied with one-half-strength Broughton and Dilworth (B&D) nutrient solution. Plants were inoculated with *Mesorhizobium loti* strain MAFF303099 when the first true leaf appeared. In contrast to the typical root hair curling phenotype of WT plants (Figure 3A), the root hairs of the T1 transgenic plants exhibited excessive swelling and branching after 3~6 days post inoculation with *M. loti* - a similar phenotype to that of the mutant EMS61 that contains loss of function *SYMRK* mutation (Figures 3B–D; Stracke et al., 2002). After 12 days post inoculation, we found no distinguishable nodule primordium or nodules formed on the mutant roots (Figures 3F–H) compared with the normal nodules produced by WT roots (Figure 3E).

### CRISPR/Cas9 induced indels and large DNA fragment deletions of *LjLb1/2/3* genes in hairy root transformed lotus plants

**Figure 4:**
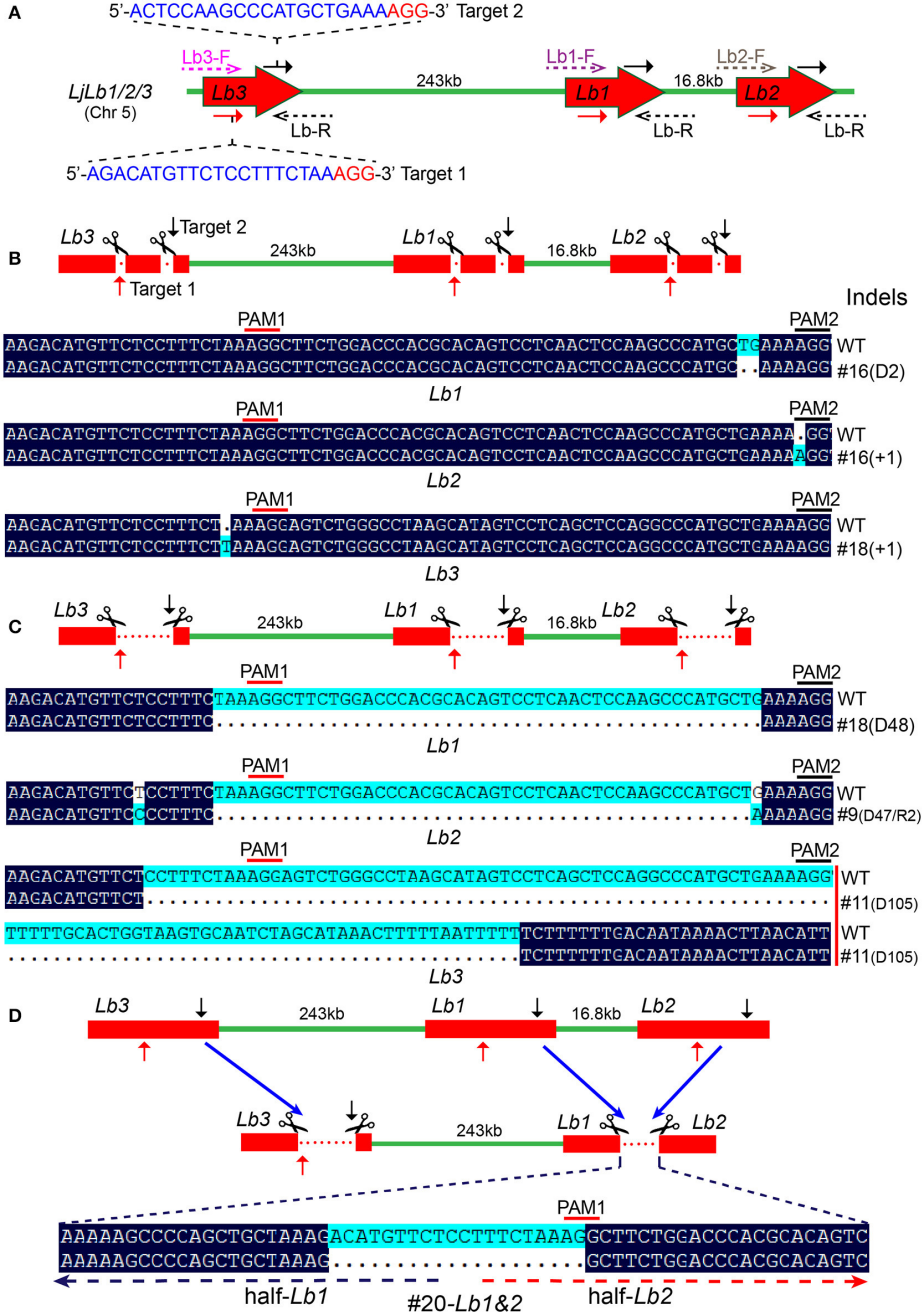
**Simultaneous disruption of 3 leghemoglobin genes (*LjLb1/2/3*) with 2 sgRNAs by hairy root transformation in Lotus. (A)** Schematic diagram illustrating the distribution and physical map of three *LjLb* genes on chromosome 5. The sgRNA targets are highlighted in blue and the PAM sequences are in red. PCR amplifications spanning the target loci were performed with gene specific primers (Lb1-F, Lb2-F, Lb3-F, respectively) and a common reverse primer (Lb-R). **(B)** Typical small Cas9/sgRNA-dependent indel modifications obtained at identical sgRNA target sites shared by the three Lotus Lbs genes in transgenic plants #16-*Lb1*, #16-*Lb2* and #18-*Lb3*. **(C)** Moderate-sized DNA deletions between two sgRNAs target sites or at a single sgRNA target site. The deletions ranged from < 50 bp to more than 100 bp. More than 30% of transgenic hairy roots contained this type of mutation. **(D)** Large fragment deletion between *LjLb1* and *LjLb2* genes of plant #20. The two sgRNA targets, 1 and 2, in each *Lbs* gene were marked with a red and a black arrow, respectively. The PAM1 and PAM2 regions were highlighted in red and black bars, respectively. D, DNA deletion; R, DNA replacement; +1, 1 bp insertion.

The Lotus genome encodes three leghemoglobin genes with high sequence similarity. The three *LjLb* genes are all located on chromosome 5 (Figure 4A). *LjLb*-RNAi plants formed white nodules instead of leghemoglobin-rich pink nodules (Ott et al., 2005). Hence, the abundance of white nodules can be an easily scorable phenotype with which to estimate the efficiency of multi-gene modifications caused by CRISPR/Cas9. We transformed *A. rhizogenes* LBA1334 with the binary vector CRISPR-LbsgRNA1&2 and used these bacteria to obtain hairy root transformants of Lotus. LbsgRNA 1 targets identical sites (target site 1) in all three *LjLbs* genes (Figure 4A). Likewise, LbsgRNA 2 targets identical sites in *LjLb1* and *LjLb2* (target site 2) which is only 48 bp downstream of target site 1 (Figure 4A). The target site 2 on *LjLb3* has two mismatches against the sgRNA 2 (Table S3).

70 transgenic plants were analyzed and 20 of them (~29%) formed white nodules after inoculated with *M. loti*. Genomic DNA was extracted and PCR was executed with gene specific forward primers (Lb1/2/3-F) and a common reverse primer (Lb-R) to determine mutation types. Unexpectedly, we found a mixture of simple and complex mutations in these plants, including deletions (representative plants #16, #18, #9, #11; Figures 4B,C, Figure S4A), insertions (representative plants #16, #18; Figure 4B), substitutions (representative plants #9, #11; Figure 4C, Figure S4A) and large fragment (~17 kb) deletion between *LjLb1* and *LjLb2* in plant #20 (Figure 4D). The moderate sized deletions in plants #18 and #9 (Figure 4C) appear to be rather precise deletions of the DNA sequences between the target site 1 and target site 2 of Cas9 cleavage sites in the *Lb1* gene and the *Lb2* gene, respectively.

**Figure 5:**
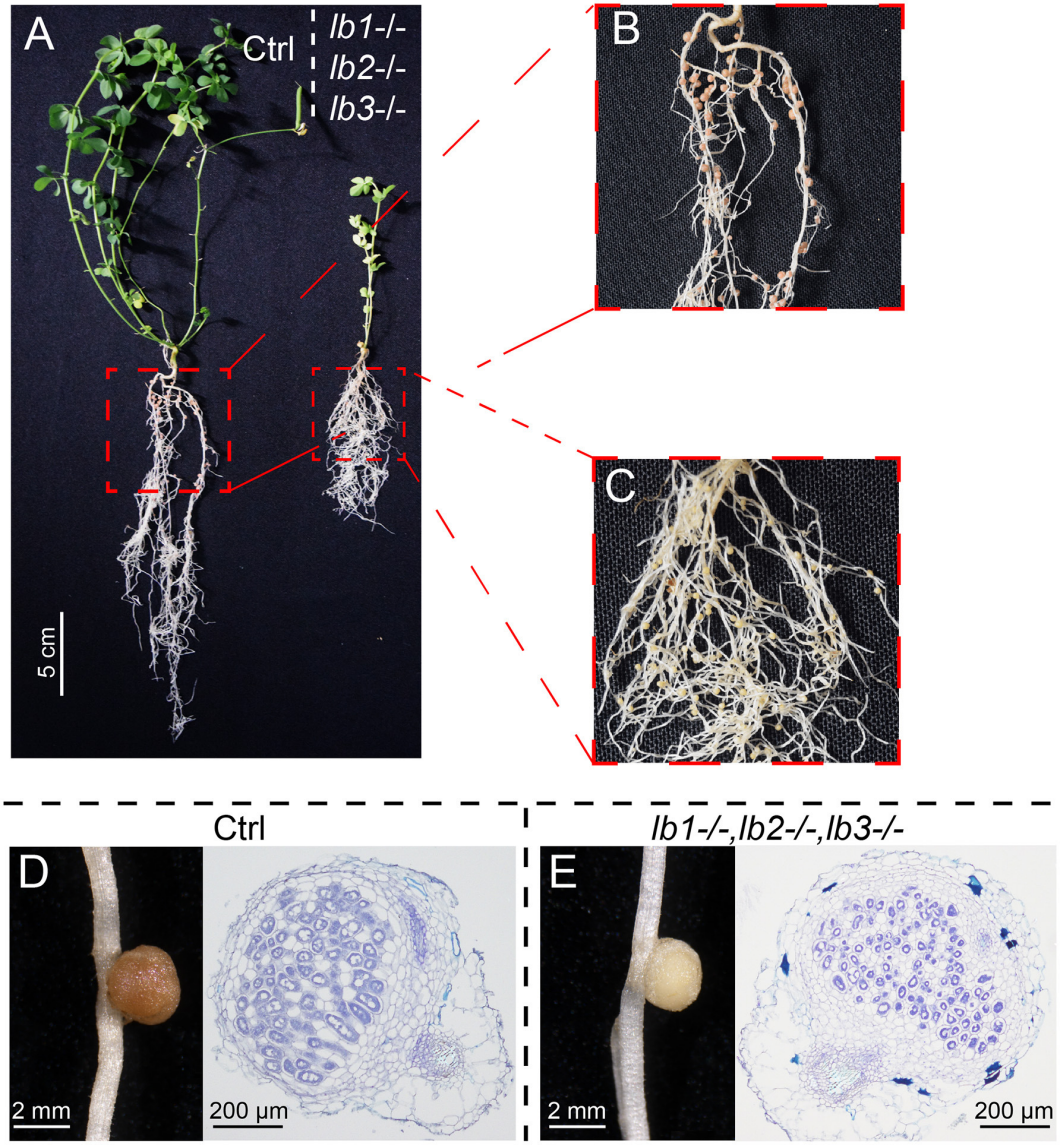
**Symbiotic phenotypes of *LjLb1/2/3* triple mutant. (A)** Under nitrogen deficient conditions, control plants (Ctrl) transformed with the empty vector exhibited robust vegetative growth with pink nodules **(B)** 8 weeks post inoculation with *M. loti*, whereas the hairy-root-transformation derived triple mutant plant #11 containing knockouts of all three *LjLbs* genes displayed reduced shoot growth with white nodules **(C)**. **(D)** and **(E)** show distinctive colors and symbiosome morphology of nodules from wild type plants and the triple mutant, respectively.

Phenotypic analysis was performed on the representative triple mutant plant #11 (Figure 5), which exhibited significant reductions in shoot and root mass compared with a control plant harboring an empty CRIPSR vector 8 weeks post inoculation (Figure 5A). Control plants formed pink nodules (Figure 5B), while the triple mutant plant #11 produced only white nodules (Figure 5C). Moreover, the symbiosomes of plant #11 showed abnormal shape, seemed smaller and degraded compared with the radial-distributed, rod-shaped bacteroids and the regular-sized symbiosomes in control plants (Figures 5D,E). Overall, plant #11 demonstrated quite similar phenotypes to *LjLb1/2/3* RNAi transgenic plants (Ott et al., 2005, 2009). DNA sequencing confirmed that all three *LjLb*s contained DNA deletions, i.e., a 105 bp deletion in *LjLb3* and, as noted above, a 48 bp deletion in *LjLb1* and a 47 bp deletion and 1 bp substitution in *LjLb2* (Figure S4A). Immunoblot analysis also confirmed the absence of LjLb1/2/3 proteins in the white nodules of plant #11 (Figure S4B).

### CRISPR/Cas9 induced disruption of *LjLb1/2/3* genes in stable transgenic lotus plants

We also transformed *A. tumefaciens* EHA105 with the binary vector CRISPR-LbsgRNA1&2 and obtained stable transgenic lines. We have confirmed five *ljlb* mutants from ~135 T1 plants, with various mutation types of the three *LjLb* genes (Figure S5A). T2 generation plants of the *LjLb* triple mutant line3-A7 was assayed for the symbiotic phenotype. After 4 weeks post inoculation, vegetative growth of the line 3-A7 was significantly inhibited, with apparent chlorosis in the leaves, indicating the nitrogen deficiency phenotype (Figure S5B). Consistently, the triple mutant plants have small and white nodules caused by the absence of leghemoglobins (Figure S5C). In contrast, the MG20 WT plants form large and pink nodules which are effective in symbiotic nitrogen fixation (Figure S5D).

### Nodule-specific expression of Cas9 confers similarly efficient gene modifications in transgenic hairy roots

**Figure 6:**
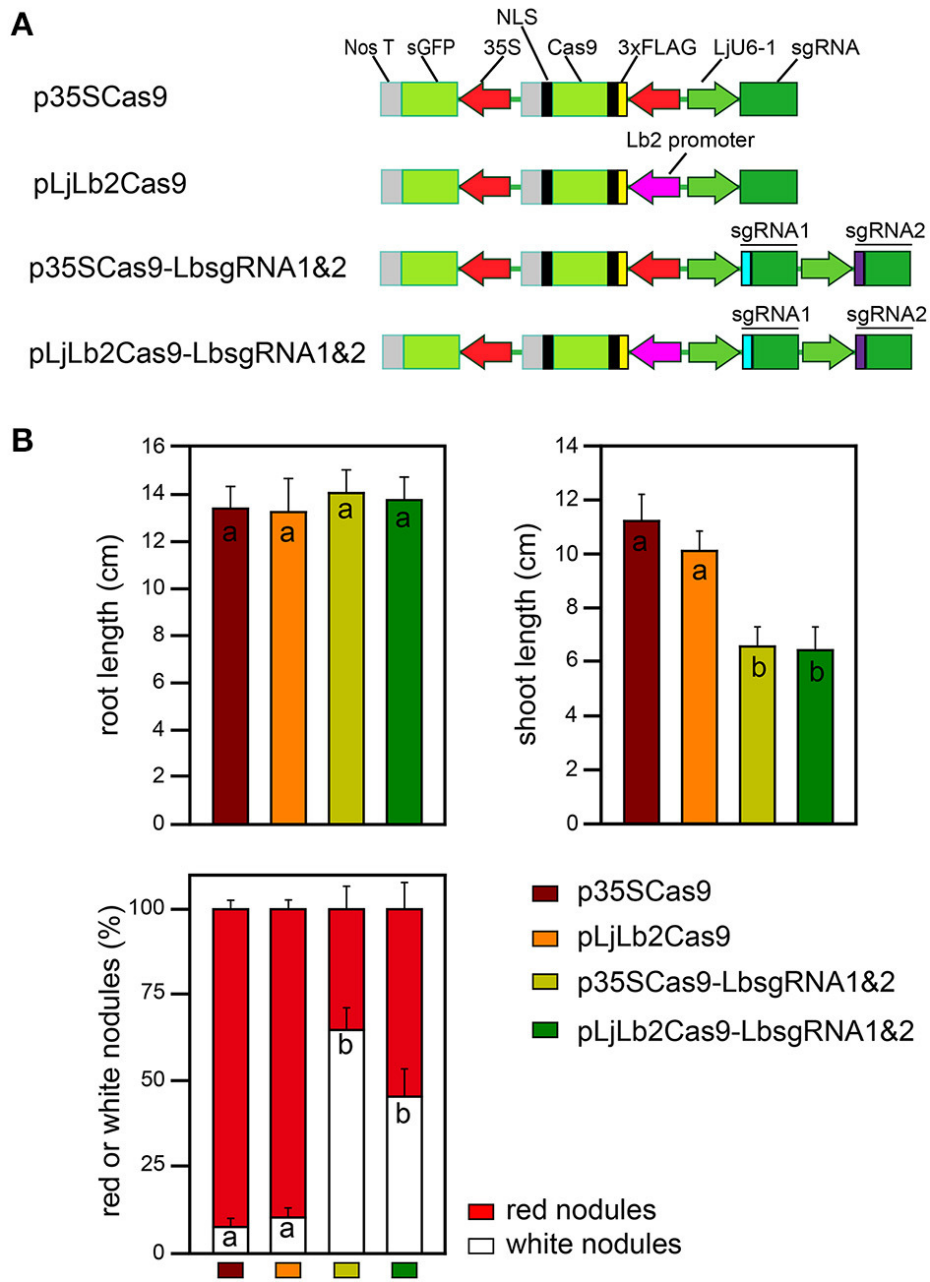
**Nodule-specific *LjLb2* gene promoter-mediated knockout of *LjLb* genes in transgenic hairy roots. (A)** Diagram of various CRISPR/Cas9 constructs used for hairy root transformation. The Cas9 cassette was driven by the 2 × 35S gene promoter (red) or *LjLb2* gene promoter (magenta). **(B)** Root length, shoot length and percentage of white or red nodules were analyzed 4 weeks after inoculation with *M. loti*. Vertical bars represent the SD of the mean (*n* > 20 in each genotype). Different letters (a or b) indicate that mean values are significantly different among various hairy root transgenic plants (*p* < 0.01, Student's *t*-test).

Constitutive promoters such as CaMV 35S, ubiquitin or actin promoters, are the most commonly used promoters to express Cas9 nuclease in various plants. However, these promoters occasionally cannot maintain relatively high level of gene expression in specific tissues (Feng et al., 2014). To compare the efficiency of nodule specific *LjLb* gene promoters in driving Cas9 expression for targeted gene modifications, we replaced the CaMV 35S promoter with the *LjLb2* gene promoter (~1400 bp long) (Figure 6A). Four weeks after inoculation with *M. loti*, we found the percentage of white nodules of transgenic roots expressing p35SCas9-LbsgRNA1&2 (65 ± 6%) and of roots expressing pLjLb2Cas9-LbsgRNA1&2 (45 ± 7%) were both significantly increased (*p* < 0.01, Student's *t*-tests) compared with the respective control lines lacking sgRNA1&2 gene constructs (p35SCas9, 7 ± 2%; pLjLb2Cas9, 10 ± 2%; Figure 6B, bottom panel). The root lengths were similar among all four types of transgenic plants (Figure 6B, upper left panel). Consistent with the increased percentage of nitrogen-fixation-deficient white nodules in transgenic roots containing sgRNA1&2 gene constructs, the two types of corresponding transgenic plants showed reduced shoot length under nitrogen deficient conditions (Figure 6B, upper right panel).

## Discussion

Compared with other precise genome editing technologies such as ZFNs and TALENs, the CRISPR/Cas9 system comprises only two simple parts, a CRISPR-associated protein 9 nuclease and a customizable single guide RNA (sgRNA) that specifies the target DNA sequence in the genome. In this study, we chose the Lotus endogenous *U6* promoter to express sgRNA rather than using Arabidopsis *U6* promoters because the transcriptional efficiency of different *U6* promoters can be quite different (Li et al., 2007). In a recent study in soybean by Sun et al. (2015), the genome editing efficiency of the CRISPR/Cas9 system using the native *GmU6-10* promoter to drive the sgRNA gene was 1.8~6.3-fold higher than when the *AtU6-26* gene promoter was used. Our results demonstrate that the CRISPR/Cas9 system can support efficient editing of single gene or simultaneous editing of multiple genes in *Lotus japonicus*. For single gene editing, we chose a single sgRNA to modify *LjSYMRK* loci and observed 35% of plants with the target gene mutated. The relatively high gene editing efficiency we obtained could be attributed to the use of a native Lotus U6 gene promoter in our customized sgRNA design and to a longer period of transgenic plants selection, i.e., an extension of the calli selection stage for additional 2–3 rounds.

It has been shown that by using 1 sgRNA, small deletions were the most common type of mutations produced in plants, while two or more target sites in single genes could lead to deletion of the fragments between the target sites (Ma et al., 2015). The higher efficiency of complete gene knock-out in rice by targeting one gene with two guide RNAs was also observed by Xie et al. (2015). By using a tandem array of tRNA-gRNA architecture, Xie et al. (2015) could assemble up to 8 sgRNAs in the same vector and achieved successful editing of four MAPK genes simultaneously in both rice protoplasts and stable transgenic plants. In our study, we found several cases of single nucleotide substitutions or 3 (or 6) nucleotide deletions by using 1 sgRNA to disrupt *LjSYMRK* (i.e., Line #1, Line #4, Line#8, Line #12, Line #15), which theoretically resulted in no mutation or 1 or 2 amino acids deletion of the protein. In contrast, we found moderate to large fragment deletions of the three highly conserved *LjLbs* genes by 2 sgRNAs. Thus, we recommend designing at least 2 sgRNA for the same gene to increase the gene knock-out efficiency and also for high throughput PCR band shift identification.

There are a number of available technological improvements to further optimize the CRISPR/Cas9 system regarding to increasing the specificity and higher efficiency. Whole genome sequencing analysis in plants have uncovered low to negligible mutations at off-target sites compared to animal systems (Zhang et al., 2014). However, the degree to which off-target mutations take place in plants still needs to be systematically investigated. In our case, Although the sgRNA2 sequence had two mismatches against the 14 and 20th nucleotide upstream of PAM region of *LjLb3* gene (Table S3), the two mismatches did not reduce the *LjLb3* gene editing efficiency significantly. We still observed disruption of *LjLb3* gene around this recognition site in both hairy root transgenic plant #11 (Figure S4) and the stable transgenic line 3-A7 (Figure S5). Similar observations have also been reported by others. For example, Cong et al. (2013) found that a single-nucleotide mismatch located 13 bp 5′ upstream of PAM still retained activity against the human *EMX1* locus. In this regards, novel strategies for modifying sgRNA genes (e.g., Doench et al., 2016), Cas9 cleavage strategies (e.g., Ran et al., 2013), mutations designed to create specific structural modifications of Cas9 and its affinity for the target DNA (Doench et al., 2016; Kleinstiver et al., 2016; Slaymaker et al., 2016) and publicly available bioinformatics tools (Belhaj et al., 2015) can be combined to enable more efficient sgRNA design and achieve higher-specificity in knocking-out genes in *Lotus japonicus*.

In this study, we confirmed the CRISPR/Cas9 genome editing ability in *Lotus japonicus* by both hairy root transformation and stable transformation. We can not directly compare the editing efficiency between the two different transformation methods, since single and multiple CRISPR events were carried out by different approaches. Transient hairy root transformation can be adopted to rapidly test gene functions in these legumes. In contrast, stable transformation is a lengthy process, requiring 4~6 months to produce transgenic lines, but the genetic background is stable and the phenotypes are more consistent. Based on our experience, combining these two systems together is powerful for loss of function analyses of genes that function in roots and root nodules.

A few symbiotic nitrogen fixation-related genes have been identified over the last two decades by screening the available mutant libraries of model legumes, especially *Lotus japonicus* (Yano et al., 2008) and *Medicago truncatula* (Smit et al., 2005). More detailed analysis of the biological functions of these genes and their interacting partners would require sophisticated and efficient targeted mutagenesis in the model legumes. As an effective supplement to the available EMS, *Tnt1* and *LORE1* retrotransposon mutant libraries, the CRISPR/Cas9 technology has the potential to make genome editing a routine practice in Lotus and significantly shorten the time to acquire mutants of multiple genes, especially the closely related genes with high homologies.

In summary, our findings demonstrate that the CRISPR/Cas9 system can effectively induce mutations in SNF related genes in *Lotus japonicus*. We predict this technology will significantly advance the speed and quality of investigations into the molecular mechanisms of nodulation and nodule function in Lotus and other legumes.

## Author contributions

LxW and DD designed the research and analyzed the results. LxW, LlW, and QT performed the experiments. LxW and DD wrote the paper. All the authors read and approved the manuscript.

### Conflict of interest statement

The authors declare that the research was conducted in the absence of any commercial or financial relationships that could be construed as a potential conflict of interest.

## Supplementary material

Figure S1 **Sequence alignment of the *U6* genes of *Lotus japonicus*. (A)** Alignment of Arabidopsis *U6-26* and 9 Lotus *U6* genes. Upstream sequence element (USE), TATA-box and *U6* small nuclear RNA (snRNA) sequences were underlined. **(B)** Sequence of the sgRNA expression cassette. The *LjU6-1* gene promoter and sgRNA were highlighted in red and blue, respectively. The *Bbs*I site in *LjU6-1* gene promoter was mutated (G → C) and underlined.

Figure S2 **Expression of sgRNA and Cas9 in tobacco. (A)** Reverse transcription PCR (RT-PCR) test of sgRNA expression. CRISPR vectors containing different sgRNAs were transiently expressed in tobacco leaves. RNA samples were also treated with RNase as negative RT-PCR controls. **(B)** Immunoblot analysis of Cas9 protein accumulation in tobacco leaves by using a FLAG antibody. Protein extract from wild type *N. benthamiana* leaves was used as a negative control.

Figure S3 **Analysis of *LjSYMRK* gene expression in the stable transgenic lines. (A)** RT-PCR assay of the *symrk* mutant EMS61, the two CRISPR knock-out lines #18 and #20, with wild-type MG20 as control. Primers SYMRK-RT-F and SYMRK-RT-R were designed to target exon 2 and exon 5 of *SYMRK* gene, respectively. The sequencing chromatographs of RT-PCR products are oriented from 3′ to 5′. Pink arrowheads indicate the 2 bp deletion sites. **(B)** Assay of relative expression levels of *SYMRK* by qRT-PCR using primers SYMRK-qRT-F and SYMRK-qRT-R. Total RNA was extracted from roots of three plants of each line 5 days post inoculation with *M. loti* MAFF303099. Ubiquitin (*LjUBI*) gene was used as an endogenous control.

Figure S4 **Characterization of the hairy root transgenic *LjLb1/2/3* triple mutant plant #11. (A)** Gene mutation types of the three *LjLbs* genes in plant #11. The two sgRNA target sites, 1 and 2, were marked with red and black arrows, respectively. The two PAM sites, 1 and 2, likewise, were labeled with red and black bars. D47/48/105, 47 bp/48 bp/105 bp DNA deletions; R1, 1 bp replacement. Pink arrowheads indicate the mutation sites in each of the three *LjLbs* genes. The sequencing chromatographs are oriented from 3′ to 5′. **(B)** Immunoblot analysis of LjLbs protein (expected molecular weight ~15 kD) accumulation in red nodules of roots of control plants (WT) and lack of accumulation in the white nodules of the transgenic hairy roots of triple mutant plant #11 (CRISPR-Lb1/2/3), using antibody against soybean leghemoglobin (GmLba). *E.coli* expressed and purified GST-Lb3 protein (~41 kD) was used as a positive control.

Figure S5 **Characterization of *LjLbs* stable transgenic lines. (A)** Mutation types of the stable transgenic T1 plants. Each *LjLb* gene was amplified by PCR and sequenced to verify the mutations. **(B)** Nitrogen-deficiency phenotype of T2 plants of the *LjLbs* triple mutant Line 3–A7. Lotus plants were inoculated with *M. loti* MAFF303099 and the phenotype was compared after 4 weeks post inoculation. Scale bar, 2 cm. **(C)** The triple mutant Line3–A7 formed small and white nodules, whereas large and pink nodules were observed in WT plant **(D)**. Scale bar, 1 cm.

Table S1 **List of primers used in this study**.

Table S2 **List of plasmids used in this study**.

Table S3 **Sequences of target genes**.
