# Supplementary figures and images for: Efficient Inactivation of Symbiotic Nitrogen Fixation Related Genes in Lotus japonicus Using CRISPR-Cas9
Source: Front Plant Sci. 2016 Aug 31;7:1333. doi: 10.3389/fpls.2016.01333 (PMC5006320; doi:10.3389/fpls.2016.01333)

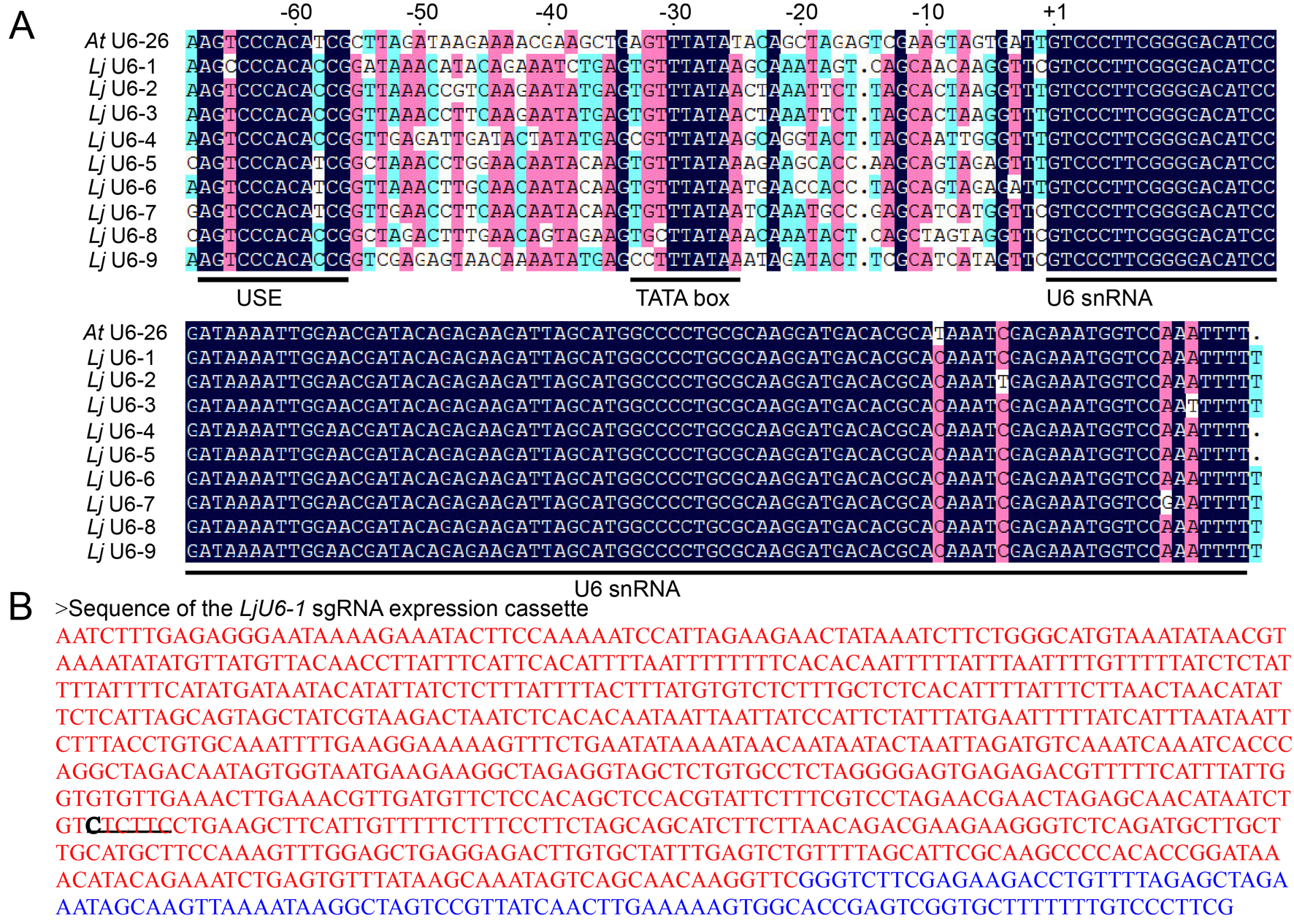

Supplement: Figure S1 — Sequence alignment of the U6 genes of Lotus japonicus. (A) Alignment of Arabidopsis U6-26 and 9 Lotus U6 genes. Upstream sequence element (USE), TATA-box and U6 small nuclear RNA (snRNA) sequences were underlined. (B) Sequence of the sgRNA expression cassette. The LjU6-1 gene promoter and sgRNA were highlighted in red and blue, respectively. The BbsI site in LjU6-1 gene promoter was mutated (G → C) and underlined. [file Image1.TIF]

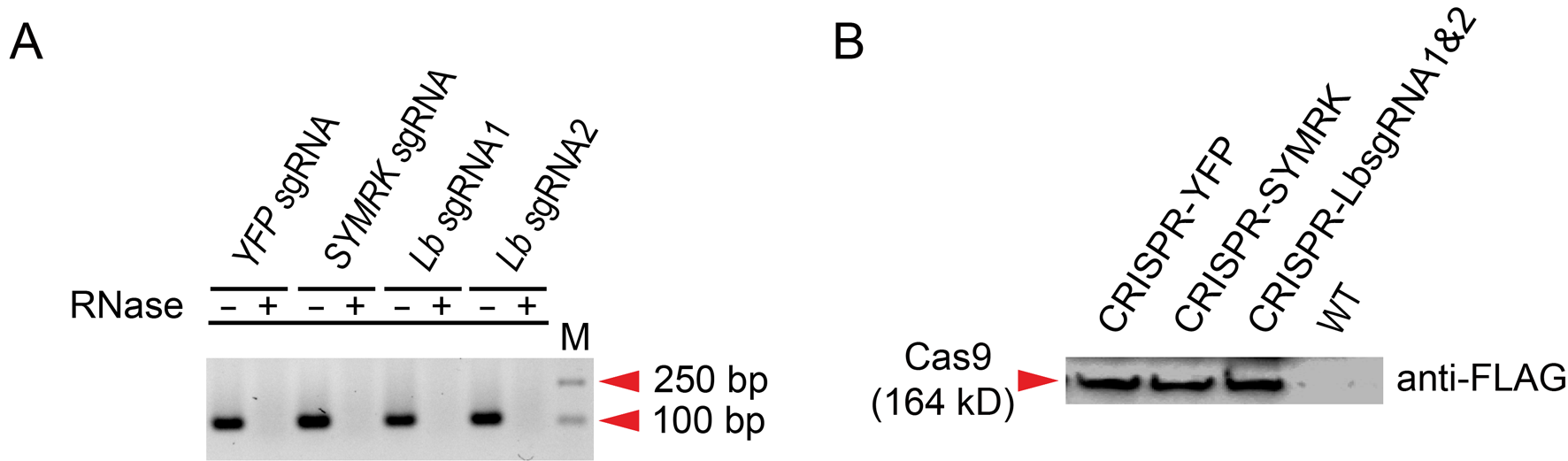

Supplement: Figure S2 — Expression of sgRNA and Cas9 in tobacco. (A) Reverse transcription PCR (RT-PCR) test of sgRNA expression. CRISPR vectors containing different sgRNAs were transiently expressed in tobacco leaves. RNA samples were also treated with RNase as negative RT-PCR controls. (B) Immunoblot analysis of Cas9 protein accumulation in tobacco leaves by using a FLAG antibody. Protein extract from wild type N. benthamiana leaves was used as a negative control. [file Image2.TIF]

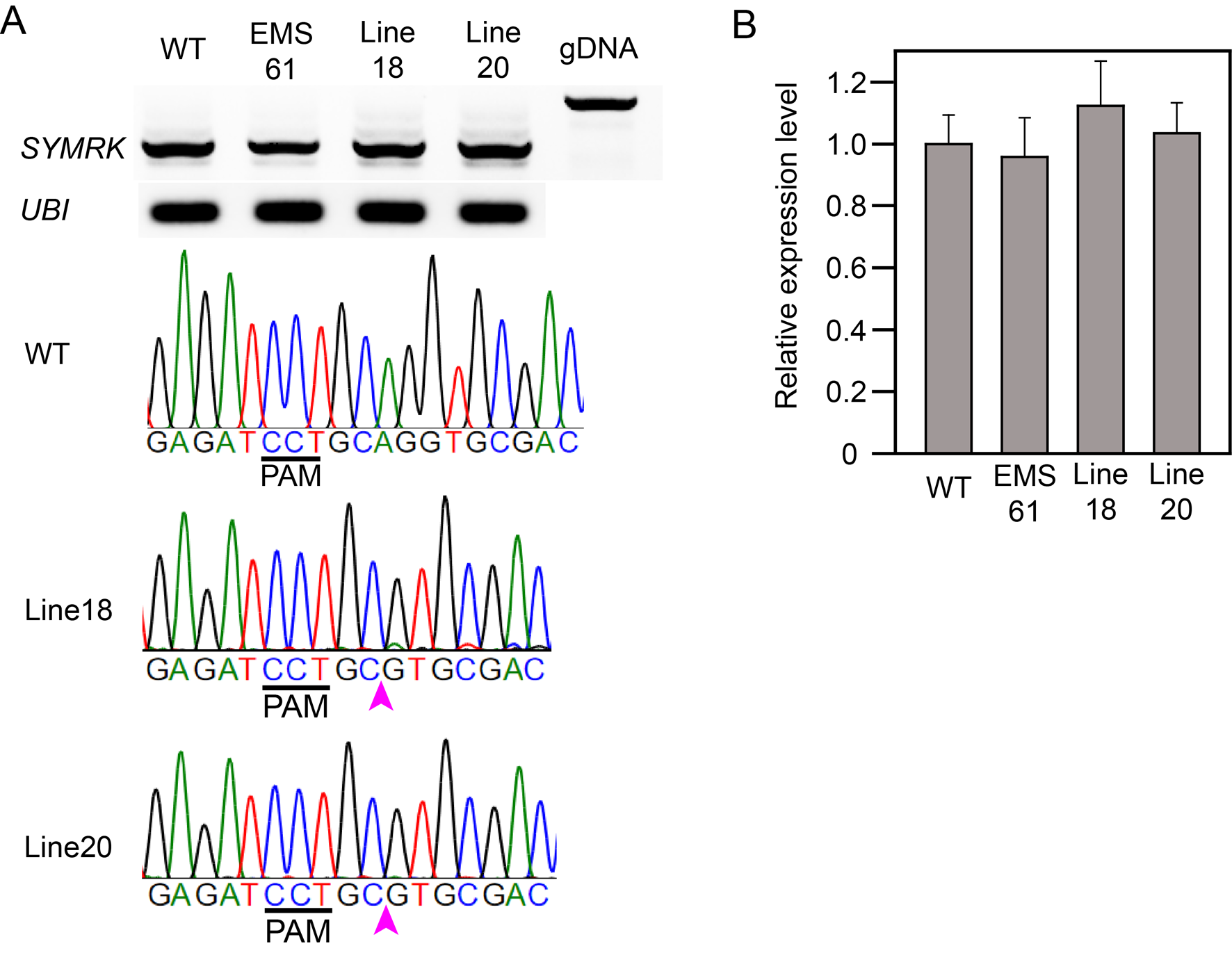

Supplement: Figure S3 — Analysis of LjSYMRK gene expression in the stable transgenic lines. (A) RT-PCR assay of the symrk mutant EMS61, the two CRISPR knock-out lines #18 and #20, with wild-type MG20 as control. Primers SYMRK-RT-F and SYMRK-RT-R were designed to target exon 2 and exon 5 of SYMRK gene, respectively. The sequencing chromatographs of RT-PCR products are oriented from 3′ to 5′. Pink arrowheads indicate the 2 bp deletion sites. (B) Assay of relative expression levels of SYMRK by qRT-PCR using primers SYMRK-qRT-F and SYMRK-qRT-R. Total RNA was extracted from roots of three plants of each line 5 days post inoculation with M. loti MAFF303099. Ubiquitin (LjUBI) gene was used as an endogenous control. [file Image3.TIF]

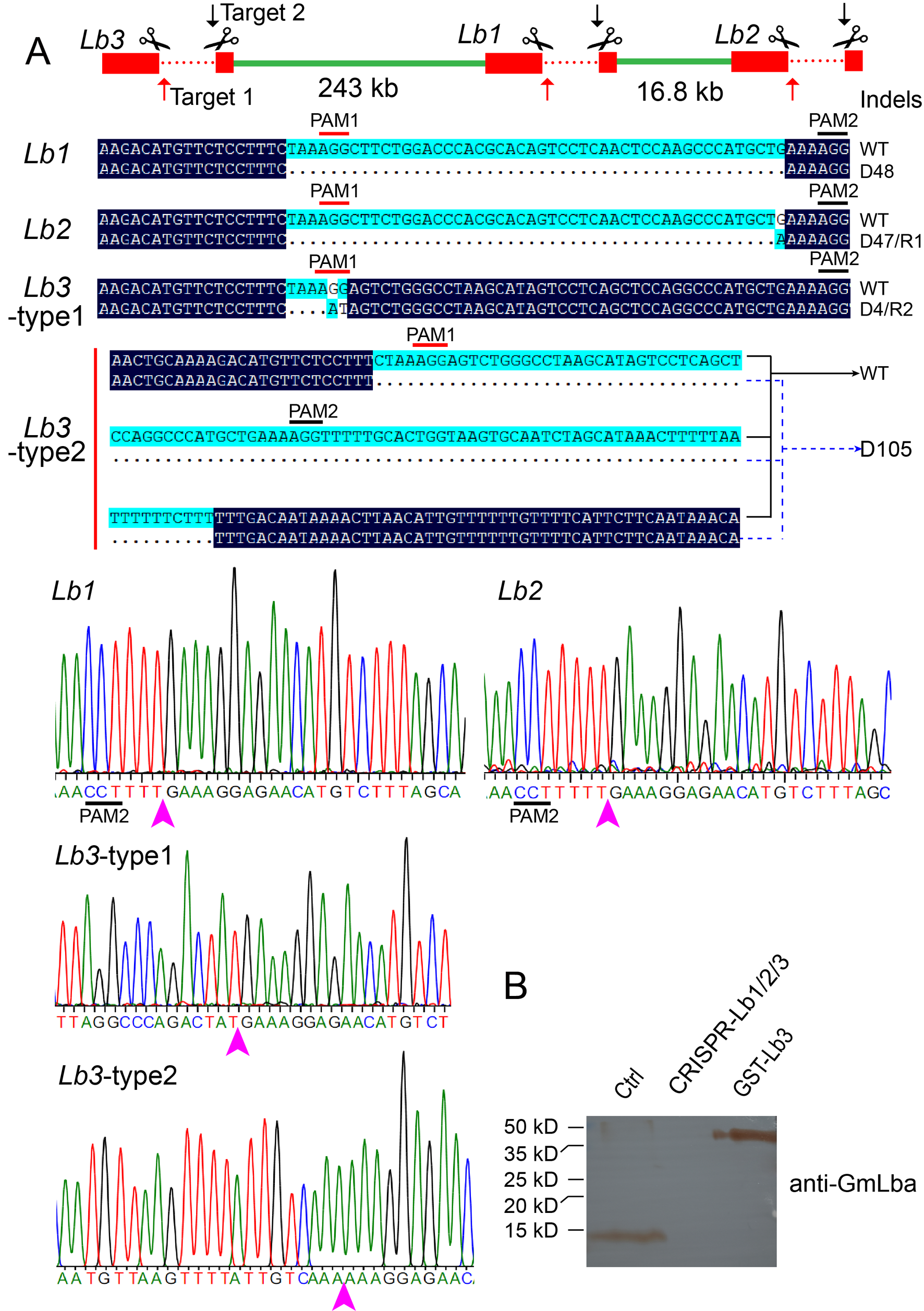

Supplement: Figure S4 — Characterization of the hairy root transgenic LjLb1/2/3 triple mutant plant #11. (A) Gene mutation types of the three LjLbs genes in plant #11. The two sgRNA target sites, 1 and 2, were marked with red and black arrows, respectively. The two PAM sites, 1 and 2, likewise, were labeled with red and black bars. D47/48/105, 47 bp/48 bp/105 bp DNA deletions; R1, 1 bp replacement. Pink arrowheads indicate the mutation sites in each of the three LjLbs genes. The sequencing chromatographs are oriented from 3′ to 5′. (B) Immunoblot analysis of LjLbs protein (expected molecular weight ~15 kD) accumulation in red nodules of roots of control plants (WT) and lack of accumulation in the white nodules of the transgenic hairy roots of triple mutant plant #11 (CRISPR-Lb1/2/3), using antibody against soybean leghemoglobin (GmLba). E.coli expressed and purified GST-Lb3 protein (~41 kD) was used as a positive control. [file Image4.TIF]

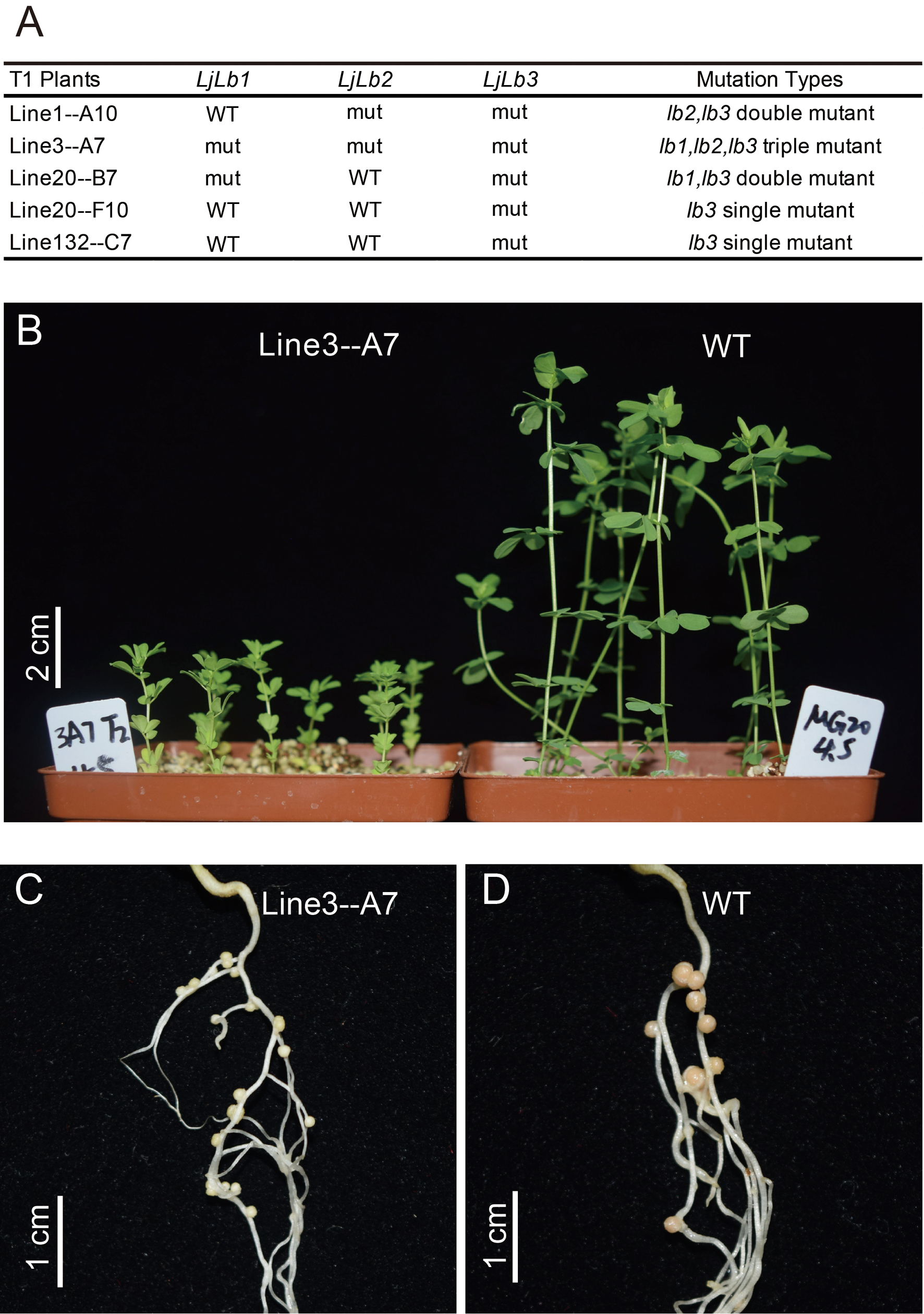

Supplement: Figure S5 — Characterization of LjLbs stable transgenic lines. (A) Mutation types of the stable transgenic T1 plants. Each LjLb gene was amplified by PCR and sequenced to verify the mutations. (B) Nitrogen-deficiency phenotype of T2 plants of the LjLbs triple mutant Line 3–A7. Lotus plants were inoculated with M. loti MAFF303099 and the phenotype was compared after 4 weeks post inoculation. Scale bar, 2 cm. (C) The triple mutant Line3–A7 formed small and white nodules, whereas large and pink nodules were observed in WT plant (D). Scale bar, 1 cm. [file Image5.TIF]
