# Supplementary material for: Efficient Inactivation of Symbiotic Nitrogen Fixation Related Genes in Lotus japonicus Using CRISPR-Cas9
Source: Front Plant Sci. 2016 Aug 31;7:1333. doi: 10.3389/fpls.2016.01333 (PMC5006320; doi:10.3389/fpls.2016.01333)
Supplement: Table S1 — List of primers used in this study. [file Table1.DOCX]

| Supplemental Table S1. List of primers used in this study | | |
| --- | --- | --- |
| Purposes | Primer Name | Primer sequence (5’-3’) |
| Cloning | SgRNA-Kpn1-Spe1-F | 5'-GGGGTACCCCGGACTAGTAATCTTTGAGAGGGAATA-3' |
|  | SgRNA-Xba1-Sal1-R | 5'-GCTCTAGAGCGTCGACCGAAGGGACAAAAAAAGCAC-3' |
|  | U6-sgRNA-fusion-F | 5'-ATAGTCAGCAACAAGGTTCGGGTCTTCGAGAAGACCT-3' |
|  | U6-sgRNA-fusion-R | 5'-AGGTCTTCTCGAAGACCCGAACCTTGTTGCTGACTATT-3' |
|  | Bbs1-mut-F | 5'-ACTAGAGCAACATAATCTGTCTCTTCCTGAAGCTTCATTG-3' |
|  | Bbs1-mut-R | 5'-CAATGAAGCTTCAGGAAGAGACAGATTATGTTGCTCTAGT-3' |
|  | Xho1-sGFP-F | 5'-ACCTCGAGGATCCCATGGTGAGCAAG-3' |
|  | Xho1-sGFP-R | 5'-TATGGAGAAACTCGAGCC-3' |
|  | LjLb2-pro-1363-KpnI-F | 5'- GTTGGTACCCAATAATTATTGTCG-3' |
|  | LjLb2-pro-24-Xho1-R | 5'-CCGCTCGAGTTTTCTTTTTTGTTTCCTTTTTG-3' |
| sgRNA | YF-FP-sgRNA-F | 5'-GATTGTGAACTTCAAGATCCGCCA-3' |
|  | YF-FP-sgRNA-R | 5'-AAACTGGCGGATCTTGAAGTTCAC-3' |
|  | SYMRK-sgRNA-F | 5'-GTTCGCAATCCACGGTCGCACCTGC-3' |
|  | SYMRK-sgRNA-R | 5'-AAACGCAGGTGCGACCGTGGATTGC-3' |
|  | Lb-sgRNA1-F | 5'-GTTCGAGACATGTTCTCCTTTCTAA-3' |
|  | Lb-sgRNA1-R | 5'-AAACTTAGAAAGGAGAACATGTCTC-3' |
|  | Lb-sgRNA2-F | 5'-GTTCGACTCCAAGCCCATGCTGAAA-3' |
|  | Lb-sgRNA2-R | 5'-AAACTTTCAGCATGGGCTTGGAGTC-3' |
| PCR-RE and sequencing | SYMRK-F | 5'-CTGTTCCTCTGTCTTCCAA-3' |
|  | SYMRK-R | 5'-ATGGGAGCAGAATACAAGG-3' |
|  | Lb1-F | 5'-TCTTGGACCTTTCTCTCCCTAATC-3' |
|  | Lb2-F | 5'-GATTTCTTAACACGTACGTATTCTTG-3' |
|  | Lb3-F | 5'-GATCTGTAGAACTCAGGACTC-3' |
|  | Lb-R | 5'-GCATCTGCAAGTGTCACTTCTC-3' |
| RT-PCR and qRT-PCR | UBI-F | 5'-TTCACCTTGTGCTCCGTCTTC-3' |
|  | UBI-F | 5'-AACAACAGCACACACAGACAATC-3' |
|  | SYMRK-RT-F | 5'-TGCAGTGAGATCATCCAGGCTC-3' |
|  | SYMRK-RT-R | 5'-TCTAAGTTGGTCATCTCAGCAATGCTGG-3' |
|  | SYMRK-qRT-F | 5'-TGAATGGAATTCTTTTGATTCG-3' |
|  | SYMRK-qRT-R | 5'-TCACTTGCACTTGACCCAGA-3' |
|  | RT-sgRNA-R | 5'-CGAAGGGACAAAAAAAGCAC-3' |
