# Supplementary material for: Efficient Inactivation of Symbiotic Nitrogen Fixation Related Genes in Lotus japonicus Using CRISPR-Cas9
Source: Front Plant Sci. 2016 Aug 31;7:1333. doi: 10.3389/fpls.2016.01333 (PMC5006320; doi:10.3389/fpls.2016.01333)
Supplement: Table S2 — List of plasmids used in this study. [file Table2.DOCX]

| Supplemental Table S2. List of plasmids used in this study | | |
| --- | --- | --- |
| Plasmid name | Description | Reference |
| pCAMBIA1300 | Plant binary expression vector, Hyg^r^ Kan^r^ | Invitrogen |
| pMD19T-simple | Intermediate carrier vector for cloning of target DNA product, Amp^r^ | TAKARA |
| pBluescript SK(+)-LjU6-TracrRNA | Intermediate carrier containing *ProLjU6::TracrRNA* Expression unit, Amp^r^ | This study |
| pBluescript SK(+)-2x35S-Cas9 | Intermediate carrier containing *Pro2x35s::Cas9* Expression unit, Amp^r^ | ([Feng et al., 2013](#_ENREF_1)) |
| pBluescript SK(+)-*LjLb2_pro1363_*-Cas9 | Intermediate carrier containing *ProLjLb2::Cas9* Expression unit, Amp^r^ | This study |
| pA7-YF-FP | Reporter plasmid containing YF and FP split fluorescent protein, Amp^r^ | ([Feng et al., 2013](#_ENREF_1)) |
| CRISPR-YFP | pC1300 containing Pro2x35s::Cas9, *ProLjU6::YFPsgRNA-TracrRNA*, Hyg^r^ Kan^r^ | This study |
| CRISPR-SYMRK | pC1300 containing Pro2x35s::Cas9, *ProLjU6::LjSYMRKsgRNA-TracrRNA*, Hyg^r^ Kan^r^ | This study |
| CRISPR-LbsgRNA1&2 | pC1300 containing Pro2x35s::Cas9, *ProLjU6::LjLbsgRNA1&2-TracrRNA,* Hyg^r^ Kan^r^ | This study |
| pC1300-sGFP | pC1300 with the hygromycin resistance gene replaced by sGFP from pUB-GFP, Kan^r^ | This study |
| p35SCas9 | pC1300-sGFP containing *Pro2x35s::Cas9* Expression unit, Kan^r^ | This study |
| pLjLb2Cas9 | pC1300-sGFP containing *ProLjLb2::Cas9* Expression unit, Kan^r^ | This study |
| p35SCas9-LbsgRNA1&2 | pC1300-sGFP containing *Pro2x35s::Cas9*, *ProLjU6::LjLbsgRNA1&2-TracrRNA,* Kan^r^ | This study |
| pLjLb2Cas9-LbsgRNA1&2 | pC1300-sGFP containing *ProLjLb2::Cas9,* *ProLjU6::LjLbsgRNA1&2-TracrRNA,* Kan^r^ | This study |

Feng, Z., Zhang, B., Ding, W., Liu, X., Yang, D.L., Wei, P., et al. (2013). Efficient genome editing in plants using a CRISPR/Cas system. *Cell Res* 23, 1229-1232. doi: 10.1038/cr.2013.114.
