# Supplementary material for: Efficient Inactivation of Symbiotic Nitrogen Fixation Related Genes in Lotus japonicus Using CRISPR-Cas9
Source: Front Plant Sci. 2016 Aug 31;7:1333. doi: 10.3389/fpls.2016.01333 (PMC5006320; doi:10.3389/fpls.2016.01333)
Supplement: Table S3 — Sequences of target genes. [file Table3.DOCX]

Supplemental Table S3. Sequences of target genes.

1. > *LjSYMRK* PCR target

**CTGTTCCTCTGTCTTCCAA**TGTCAGCAATGTTGATCTCAACGCCAATGTAACGCCGCCCCTAACAGTCCTACAAACAGCTCTAACCGACCCTGAGCGATTGGAGTTCATCCACACTGACCTTGAGACAGAGGATTATGGATACCGTGTGTTCCTCTACTTCCTTGAATTAGATAGAACTCTACAAGCAGGCCAAAGGGTGTTTGACATATATGTTAACAGTGAGATTAAAAAGGAGAGTTTTGATGTATTGGCTGGAGGGTCAAACTATAGATACGATGTCTTGGACATTTCAGCCAGTGGATCACTTAATGTAACCTTGGTCAAGGCATCTAAGTCTGAGTTTGGACCCCTTTTGAATGCTTATGAGATCCTGCAGGTGCGACCGTGGATTGAAGAGACAAACCAAACAGATGGTAAGCGTAGTTGAAGGTCCTAGCATCATTTATCTGTTTTACATTCCTTTTTTCTTATTCATCTCTGAACTAACTTCCCCACATTGAACCTTCAATCTTTTCTCACTCTCTTTCATTGTTACATAAAAAAATTCCCCTCTTTAGTGGGAGTGATTCAGAAGATGAGAGAAGAATTGTTGTTGCAAAACTCCGGCAATAGAGCGCTGGAGAGTTGGAGCGGAGAC**CCTTGTATTCTGCTCCCAT**

The *LjSYMRK* PCR primers and guide RNA sequence are highlighted in red and blue, respectively. The PAM sequence is underlined.

1. > *LjLb*1 PCR target

**TCTTGGACCTTTCTCTCCCTAATC**CATATGTCTCTGAGTTCTTACCACTTAAGTTATATATCTTCCAGGAATAGACATCGTTTCATAATTTACGAACTATTATATATTTTAATTAATTGAAAATCTGCTCGATTATATAAAGTTTTTGAAAAGAATATTATGATATTATCTCTTAATAATGCCAATGGCCAGCCATCCACAGACTAAAAATTATTCCAATCACCATCAATAAAAAATAAAAAAAAAAACTCCATCTATTTGCACTCTCCAAGTCTCCTATATAAATATTTATTGGATGTGAGGTTGTATCATCAGAGAACACAAATAACAAAAAGGAAAAAAAGAAAAAGAAAAATGGGTTTCACTGCACAGCAAGATGCTTTAGTGGGTAGCTCATATGAAGCATTCAAGCAAAACCTTCCTAGCAATAGTGTTCTGTTCTACACCTTGTAAGTCCTATCTCTCAGTGTGTCTTTGATTTATGTGTTTTCTCTTTTTGTTGTTTTGTTGTGTGTTGGAAACAAATGATGTAAATTAATGTGACCTCCTTTAATCTTGATTTGATTAAAAAAAGAATATTGGAAAAAGCCCCAGCTGCTAAAGACATGTTCTCCTTTCTAAAGGCTTCTGGACCCACGCACAGTCCTCAACTCCAAGCCCATGCTGAAAAGGTTTTTGGACTGGTAAGTGAAATCTAGCATAAAATTGTATTCTGTTTACATTCTTCTATCAGTTCTATGAATGCCAGTGTACTATTATGTTATATTTTTTGAACCATAGACACGCGATGCGGCTGCTCAACTCTTAGCAAAAG**GAGAAGTGACACTTGCAGATGC**

The *LjLb*1 PCR primers, guide RNA1 and guide RNA2 sequences are highlighted in red, blue and green, respectively. The PAM sequences are underlined.

1. > *LjLb*2 PCR target

**GATTTCTTAACACGTACGTATTCTTG**AAATAATTTTTGGACAACATACTTATTTAGTAGAAGATTATATCAAAGAAATTATACATTATATATAAATACTTTGAATGTGAATGTATAAAAGTTTATTTTTTAAAATTCACAACTTGTGTAAGTGTAAAAGTCTGGTATGACAAACAATGGTGCAATCCCATTTCAAATAATACAGCTTTTCAAAAGAGGCATAATATTTATATGATTAAATTTACAACTATTTTAGTTAATTGAAAAGTCATTTTGATTAAGTTTTTGAAAAGTTTATTGTCTCTTAATAAAACCAATGGCCAGCCATCCACCGATGCAGAAATTTTCCAATTAATCACGATCACCAGGAATCAAACTCCATCAAGCTATTTGCACTCTCCAAGTCTCCTATATATACATTTATTGGATGTGAAGTTGTATCATCGGAGGACACAAATAACAAAAAGGAAACAAAAAAGAAAAATGGGTTTCACTGCACAGCAAGATGCTTTAGTGGGTAGCTCATATGAAGCATTCAAGCAAAACCTTCCTAGCAATAGTGTTCTGTTCTACACCTTGTAAGTCCTATCTCTCAGTGTGTCTTTGATTTATGTGTTTTCTCTTTTTGTTGTTTTGTTGTGTGTTGGAAACAAATGATGTAAATTAATGTGACCTCCTTTAATCTTGATTTGATTAAAAAAAGAATATTGGAAAAAGCCCCAGCTGCTAAAGACATGTTCTCCTTTCTAAAGGCTTCTGGACCCACGCACAGTCCTCAACTCCAAGCCCATGCTGAAAAGGTTTTTGGACTGGTAAGTGAAATCTAGCATAAAATTGTATTCTGTTTACATTCTTCTATCAGTTCTATGAATGCCAGTGTACTATTATGCTATATTTTTTGAATCATAGACACGCGACGCGGCTGCTCAACTCTTAGCAAAAG**GAGAAGTGACACTTGCAGATGC**

The *LjLb*2 PCR primers, guide RNA1 and guide RNA2 sequences are highlighted in red, blue and green, respectively. The PAM sequences are underlined.

> *LjLb*3 PCR target

**GATCTGTAGAACTCAGGACTC**GTCTAAATATTAATCCCATTTAAAATAAAAGTAAGGCTTTTTAAGGCATAATATTCTATTGAAATCTAAATAATTTACCAATTATTTATTTTAATTAATTGAATAATTTGTTCGATGATGATTTTGAAAAGTTATTGTCTCTTAATAATGCTAATAGCCATGCACCACACACCAGAAATTCTTCCAATCACGATCTCAAGAAACACACTCCATCTATTTGCACTCTCCAAGTCGCCTATATAAACAATTGTTGGATGTGAAGTTGTTTCATACTTGCATTGGAGAATACAGATAACAAAAAGAAAAAAAGAAAAAGAAAAATGGGTTTCACTGCGCAGCAAGAGGCTCTAGTGGGTAGCTCATACGAAACATTCAAGAAAAACCTTCCTACCAACAGTGTTTTGTTCTACACCGTGTAAGTTTTATCTCTCAATGAGTGTCTTTCATTTGTGTGTTTTCTCTTCTATTGTTTGTTGTGTTTTGGAAAGAAATGATGTAAATAATGTGAGCCATTAATTTTGGCTTGATTAAAAACAGTATATTGGAGATAGCACCAACTGCAAAAGACATGTTCTCCTTTCTAAAGGAGTCTGGGCCTAAGCATAGTCCTCAGCTCCAGGCCCATGCTGAAAAGGTTTTTGCACTGGTAAGTGCAATCTAGCATAAACTTTTTAATTTTTTCTTTTTTGACAATAAAACTTAACATTGTTTTTTGTTTTCATTCTTCAATAAACACGGGTATACTATTACACTATATTTTTTGAATTATAGACTCGTGATGCTGCCACTCAACTCGTAGCAAAAG**GAGAAGTGACACTTGCAGATGC**

The *LjLb*3 PCR primers, guide RNA1 and guide RNA2 sequences are highlighted in red, blue and green, respectively. The PAM sequences are underlined. The *LjLb*3-guide RNA2 sequence has two mismatch sites that are highlighted yellow.
